# Specifics

**Published:** 1915-11

**Authors:** 


### Specifics.

Do not deride the hope of our fathers in medicine, that a specific might be found for every disease. It is a laudable, practical, humanitarian aim. Neither is it a mere chimera. We have made substantial progress toward its realization. In many instances, there is scientific foundation for regarding a remedy as specific for a given pathologic process. In many others, there is sound reason to hope for further developments. The fact that a given substance may be efficacious in more than one condition does not alter its value, although it indicates a modification of the original theory. Neither does the fact that 100% of efficiency has never been attained and probably never will be attained, interfere with the practical usefulness of a definite remedy of choice in a definite condition. A good deal of the pessimism regarding specifics has been due, not so much to the failure of the remedy as to lack of specificity of the disease names in common use. There never will be a specific for angina or pneumonia or rheumatism or dyspepsia, but there already are a good many remedies that are, according to a reasonable definition, specific for various pathologic conditions accurately distinguished in nature and correctly diagnosed.

